# Pre-pregnancy counselling for women with chronic kidney disease: a retrospective analysis of nine years’ experience

**DOI:** 10.1186/s12882-015-0024-6

**Published:** 2015-03-14

**Authors:** Kate S Wiles, Kate Bramham, Alina Vais, Kate R Harding, Paramit Chowdhury, Cath J Taylor, Catherine Nelson-Piercy

**Affiliations:** Guy’s and St Thomas’ NHS Foundation Trust, London, UK; King’s College London, London, UK; Imperial College Healthcare NHS Trust, London, UK

**Keywords:** Chronic kidney disease (CKD), Pregnancy, Counselling, Experience, Multidisciplinary

## Abstract

**Background:**

Women with chronic kidney disease have an increased risk of maternal and fetal complications in pregnancy. Pre-pregnancy counselling is recommended but the format of the counselling process and the experience of the patient have never been assessed. This study examines the experience of women with chronic kidney disease attending pre-pregnancy counselling and evaluates their pregnancy outcomes.

**Methods:**

This is a cross-sectional assessment of 179 women with chronic kidney disease attending a pre-pregnancy counselling clinic (2003–2011) with retrospective evaluation of aetiology, comorbidity, treatment and adverse pregnancy outcome compared with 277 hospital controls. It includes an analysis of descriptive data and free text content from 72 questionnaire responders.

**Results:**

65/72 (90%) of women found the clinic informative. 66 women (92%) felt that the consultation had helped them decide about pursuing pregnancy. 12 women (17%) found the multidisciplinary process intimidating. Free text comments supported the positive nature of the counselling experience, but also highlighted issues of access and emotional impact. Adverse pregnancy outcome rates were significantly higher in women with chronic kidney disease: 7/35 (20%) had pre-eclampsia (p < 0.001), 8/35 (23%) infants were small for gestational age (p < 0.001), 11/35 (31%) had preterm deliveries (<37 weeks) (p < 0.001) and 5/35 (14%) had a pregnancy loss compared with 4%, 10%, 8% and 3% of controls respectively.

**Conclusions:**

Women with a diverse range of renal disease severity and complexity attend pre-pregnancy counselling. Factors affecting pregnancy include hypertension, proteinuria and teratogenic medication. It is important to be able to inform women of the risks to them and their babies before pregnancy in order to facilitate informed-decision making. Most women with chronic kidney disease attending a pre-pregnancy counselling clinic report a positive experience.

**Electronic supplementary material:**

The online version of this article (doi:10.1186/s12882-015-0024-6) contains supplementary material, which is available to authorized users.

## Background

All pregnant women with chronic kidney disease (CKD) are at increased risk of maternal and fetal complications including pre-eclampsia, fetal growth restriction, preterm delivery, perinatal death and acceleration of maternal disease [1]. A systematic review reports that women with CKD have a two-fold increased risk of adverse fetal outcomes and a five-fold risk of an adverse maternal event [2]. These adverse events are substantially more common than for most other chronic diseases and yet women with CKD may be unaware that their condition has implications for pregnancy [3].

Meta-analysis data on pregnancy outcomes in CKD [4] have led to recommendations for evidence-based contemporaneous pre-pregnancy counselling, supported by guidelines from the Royal College of Obstetricians and Gynaecologists [5]. Furthermore, the confidential enquiry into maternal deaths in the UK recommended that pre-pregnancy counselling should be provided for women with pre-existing medical illnesses, in response to the identification of maternal mortality in women who had not received pre-pregnancy advice [6].

Pre-pregnancy counselling offers an opportunity to adjust medication, optimise blood pressure, quantify proteinuria and educate women about potential adverse events that may arise during, or develop as a consequence of pregnancy [7]. However, methods of pre-pregnancy counselling for women with CKD and its acceptability to women have never been examined. The aim of this study was to evaluate the experience of women with CKD who have received pre-pregnancy counselling at an established tertiary clinic. In addition, pregnancy outcomes of the counselled cohort women were compared with a control population delivering in the same tertiary unit in order to better inform the counselling process in the future.

## Methods

The multi-disciplinary pre-pregnancy clinic for women with CKD, including renal transplant recipients, was established at Guy’s and St. Thomas’ NHS Foundation Trust in 1999. Referrals are received from nephrologists, obstetricians and general practitioners predominantly from London and South East England. Women are seen by a consultant obstetric physician, specialist obstetrician, and a nephrologist for a combined consultation lasting 30–45 minutes. Women’s partners are invited to attend. Individualised, evidence-based counselling is given to women considering pregnancy. The potential outcomes of pregnancy, mode of delivery and the short term and long-term impact on maternal renal function are described in detail. Drug regimens are reviewed for potential teratogenicity; the risks and benefits of discontinuing drugs prior to or following conception is outlined, and recommendations for low dose aspirin prophylaxis [7] are discussed. Local or tertiary antenatal care and delivery are planned. Contraceptive counselling and advice regarding pre-conception folic acid is given. Women and their partners are offered the opportunity to ask any questions or request clarification about the counselling. A comprehensive letter is sent to the referring doctor, the general practitioner and the patient documenting the advice given.

Women’s experiences of the clinic were examined using a questionnaire developed by the authors. In 2001, the Institute of Medicine identified the different dimensions that constitute patient-centred care [8]. This framework was used to inform the structure and content of the questionnaire which was further determined by expert consensus from the service providers. The questionnaire examines women’s views regarding their involvement, extent of shared decision-making, and quality/usefulness of information and communication. A four-point response scale was used to maximise variability and avoid responses set to the midpoint [9]. Descriptive anchors rather than a scored response were recorded to prevent homogenous interpretation of the data due to interval scale assumption. The questionnaire was anonymised to encourage response and candour.

The questionnaire was piloted in 2002 and sent to 62 women. 30 women responded (48%). This confirmed that the patients were using the full range of possible answers and that the distribution was not disproportionately skewed. Invited free text responses were used to assess the adequacy of the included questions. Following this pilot, a final questionnaire was devised including 9 fixed, closed-format questions (see Additional file 1: Table S1). In addition, free text responses were invited with the lead ‘Do you have any other comments about your experience in the clinic?’ In 2011, the questionnaire was posted with a paid return envelope to the last known address of all attendees of the pre-pregnancy counselling clinic between 2003 and 2011 (n = 179). The questionnaire was sent to the women with an invitation to participate in the study including the purpose of the study, why the recipient had been chosen, the voluntary nature of participation and the anonymity of the response. Contact details were clearly stated. Informed consent was indicated by the return of the completed questionnaire. The fixed question data was analysed descriptively. The free text data was analysed using thematic content analysis methods [10]. All comments were read and key emergent themes were noted by two of the authors (KW, CT) independently. A final thematic coding framework was agreed and applied to all comments. The coding framework was informed by the Institute of Medicine dimensions of patient centred care [8] which underpinned the design of the questionnaire, but was also driven by the data itself and allowed to extend or challenge the Institute of Medicine dimensions as required.

Our hospital database was retrospectively interrogated for maternity outcome data that had previously been collected during the course of normal patient care. Pregnancy outcomes were available for women who had attended pre-pregnancy counselling and subsequently delivered at St. Thomas’ Hospital (n = 35). Outcomes included:

- Pre-eclampsia: Documented diagnosis in the hospital database
- Fetal growth restriction: <10th centile for gestational age [11]
- Preterm delivery: <37 weeks’ gestation
- Intrauterine death: >24 weeks’ gestation
- 2nd trimester miscarriage: 14 - <24 weeks’ gestation
- Pregnancy loss: 2nd trimester miscarriage or intrauterine death

Pregnancy outcomes were compared to a control cohort of 277 women delivering at our hospital over the same time period (January 2005-July 2011) previously reported elsewhere [12]. This control population was representative of a tertiary referral obstetric population with 18% prevalence of chronic disease, excluding pre-existing renal disease, hypertension and diabetes. Fisher’s exact test was used for bivariate comparison of dichotomous data.

### Ethics statement

This manuscript describes a service evaluation and did not require formal NHS Research Ethics Committee review. The study was designed to measure current care with the aim of establishing the standard of pre-pregnancy counselling for women with CKD at a single tertiary centre, with results that would not be generalizable outside the assessed area of practice. The analysis of existing data was performed by the direct care health team and subsequently anonymised.

The ethical nature of this evaluation was considered justified on the basis that there was no deviation from normal clinical practice. In addition participants were informed of the rationale for the study, the voluntary nature of the questionnaire and the anonymity of their response with informed consent being given by return of the questionnaire.

Other than the time taken to complete and return the questionnaire there was no additional burden or risk for participants. Although there are no explicit criteria for pre-pregnancy counselling, our use of the 2001 Institute of Medicine dimensions of patient care meant that this study was registered and approved as an audit at Guy’s and St. Thomas’ NHS Foundation Trust.

## Results

### Demographics and clinical presentation of women attending the clinic

179 women with CKD attended for pre-pregnancy counselling between 2003–2011. There was a trend for an increasing number of referrals over time (see Additional file 2: Figure S1). Age ranged from 21–46 years with a median of 33 years. 131 women (73%) were nulliparous.

The 179 women had 236 diagnoses (see Table 1). The median number of diagnoses per patient was 1 although some women had more than one diagnosis with the potential to impact a pregnancy (range 1–3), for example, diabetic nephropathy and subsequent transplantation, or hypertensive nephropathy with secondary glomerulosclerosis.

**Table 1:** **Diagnoses in women attending for pre-pregnancy counselling**

**Diagnosis**	Number of patients (%)*
Glomerulonepritis (not lupus)	41 (17)
Lupus nephritis	28 (12)
FSGS and other nephrotic syndromes	33 (14)
Reflux nephropathy	30 (13)
Diabetic nephropathy	20 (8)
Hypertensive nephropathy	13 (6)
Inherited/congenital renal disease	10 (4)
Renal stone disease	4 (2)
Other	3 (1)
Kidney transplant	41 (17)
Kidney-pancreas transplant	13 (6)

*236 diagnoses in 179 patients (123 patients with single diagnosis, 52 patients with 2 diagnoses and 3 patients with 3 diagnoses).

The severity of renal impairment pre-pregnancy ranged from CKD stage 1–5 (see Table 2).

**Table 2:** **CKD stage in the pre-pregnancy counselling cohort**

**CKD Stage**	**eGFR ml/min/1.73 m** ^**2**^	**Number of patients (%)**
1	>90	50 (30%)
2	60- < 90	48 (27%)
3A	45- < 60	32 (18%)
3B	30- < 45	31 (17%)
4	15- < 30	16 (9%)
5	<15 or dialysis	2 (1%)

Pre-pregnancy blood pressure records were available for 167 women (93% of cohort). 56 (34%) were normotensive without treatment (<140/90 mmHg), 89 (53%) had controlled blood pressure on anti –hypertensive medication and 22 (13%) had uncontrolled blood pressure (>140 or >90mmgHg) despite treatment. 77 women (43%) had detectable proteinuria and 18 women (10%) had a history of nephrotic-range proteinuria (>3 g/24 hours).

Medication data were available for 175 women: 85 women (49%) were counselled regarding a required change in medication for pregnancy including discontinuation and/or substitution of angiotensin receptor blockade in 46 women (26%) and mycophenolate mofetil in 33 women (19%). Other medications of relevance to pregnancy included bisphosphonates, diuretics, warfarin, statins and tetracycline.

### Questionnaire data

72 women responded to the questionnaire (response rate 40%). 65 women (90%) stated that they found the clinic informative, 66 women (92%) reported that they understood the advice given and 64 women (89%) felt that the consultation had helped them decide about pursuing pregnancy. Although 64 women (89%) felt that it was helpful to see three specialists together, 12 women (17%) felt that the presence of many doctors was intimidating.

42/72 (58%) of the respondents provided free text data. 31/42 (74%) provided positive comments, 6/42 (14%) were mixed and 5/42 (12%) were negative. Key themes were the expertise and professionalism of the multidisciplinary team, provision of information and understanding, access to services, decision-making, the emotional response to the consultation and provision of individualised care (see Table 3).

**Table 3:** **Content analysis of questionnaire free text data**

**Theme**	**Number of comments**	**Positive**	**Negative**	**Examples**
Expertise/Professionalism	23	23	0	• We felt very lucky to have been referred and have the opportunity to talk with a panel of consultants
				• It was fantastic to feel we had the best of advice in all areas and that a specialist from all areas was there
				• Three consultants were extremely well informed and we found their opinion and level of expertise both beneficial and reassuring
Information/understanding	20	18	2	• If we had any questions or didn’t understand they clarified things
				• It enabled me to understand the condition of my kidneys and to accept the problems I might be facing
				• I decided not to go through with it and I’m not sure that’s right
Access to services	13	11	2	• It felt like it took a long time to reach the right people
				• Letter was useful with both pregnancies at local hospital
Decision-making	12	10	2	• It is an important decision and it was – and still is – very reassuring to know that the decision we took was with the best information
				• If it had not been for the clinic and reassurance from consultants that it was safe to fall pregnant we probably would not have tried
Emotional response	9	4	5	• I found this quite stressful and frightening
				• The risks scared me so much
				• It made me feel safe
Individualised care	7	5	2	• I have other medical problems as well and the doctors were able to give me some very informed advice
				• All concerns were taken seriously
				• My thoughts and view were not taken into consideration

### Pregnancy outcomes

Outcome data were available for 35 women who attended pre-pregnancy counselling and subsequently delivered at St. Thomas’ Hospital. Prior to pregnancy, these women had CKD stages 1-3B. Rates of pre-eclampsia, intrauterine growth restriction, preterm birth, intrauterine death and mid-trimester miscarriage were significantly higher for women with CKD compared to our control population (see Table 4).

**Table 4:** **Pregnancy outcomes in women attending for pre-pregnancy counselling**

**Adverse outcome**	**All n = 35**	**CKD stage 1–2 n = 23**	**CKD stage 3 n = 12**	**Controls n = 277**
Pre-eclampsia	7 (20%)**	4 (17%)**	3 (25%)**	11 (4%)
Small for gestational age	8 (23%)**	6 (26%)**	2 (17%)**	29 (10%)
Preterm <37 weeks	11 (31%)**	5 (22%)**	6 (50%)**	21 (8%)
Preterm <34 weeks	4 (11%)**	2 (9%)**	2 (17%)**	4 (1%)
IUD/Mid-trimester miscarriage	5 (14%)**	3 (13%)**	2 (17%)**	7 (3%)

**p < 0.01.

## Discussion

This analysis of women attending a pre-pregnancy counselling renal clinic has identified that women had substantial comorbidity that will influence pregnancy outcomes and half were taking teratogenic medication. The majority of women found the clinic to be informative and felt that it influenced decision-making. Subsequent pregnancies had high rates of maternal and fetal complications compared with controls, despite attempts to optimise maternal heath in the pre-pregnancy setting. This supports the need for accurate and detailed information of pregnancy outcomes to be discussed prior to pregnancy, in order to facilitate informed decision-making about conception.

CKD affects up to 3% of women aged 20–39 years [13]. Between 2003 and 2011, 179 women with CKD from the South East of England were referred for pre-pregnancy counselling to our specialised clinic. There was an increase in referrals over the last decade which is likely to reflect trends of older primiparity [14], obesity [15] and advances in fertility treatment resulting in more pregnancies being complicated by CKD [16].

The need for pre-pregnancy counselling in women with CKD is well recognised by health professionals [3,5] and this is supported by our data. The majority of women were nulliparous with no obstetric history to inform assessment of their pregnancy-associated risk, therefore expert individual risk assessment based on comorbidity was necessary. Women attending pre-pregnancy counselling had complex renal diagnoses, and up to one third had a renal transplant. The severity of renal disease was diverse and nearly half had CKD Stages 3–5.

Although the severity of CKD is the major determinant of pregnancy-related risk, adverse outcome rates are higher even in women with CKD Stage 1 and normal glomerular filtration, compared to controls [3,17]. However, women with CKD Stages 1–2 have a reduced likelihood of adverse events compared to women with more advanced disease [1].

The association between hypertension and pregnancy-related complications is well recognised [18,19]. Women with hypertension have a substantially higher risk for the development of pre-eclampsia compared to normotensive women with the same glomerular filtration rate (GFR) [20], and uncontrolled hypertension in early pregnancy is associated with poor outcomes [21]. Conversely normotension is a strong predictor of good pregnancy outcomes [22]. Two-thirds of our pre-pregnancy cohort required treatment for blood pressure control and one in eight women had uncontrolled hypertension despite treatment, emphasising the importance of tailored pre-pregnancy care.

Severity of proteinuria is also associated with unfavourable pregnancy outcomes in some cohorts [23-25]. Just under half the women attending pre-pregnancy counselling had detectable proteinuria. Pregnant women with CKD are at risk of developing significant proteinuria due to increased glomerular filtration and altered renal protein handling, even when proteinuria is not present before pregnancy [26]. Women with CKD need to be informed about exacerbation of proteinuria in pregnancy and implications for thromboprophylaxis and diagnostic uncertainty in the context of pre-eclampsia.

Pre-pregnancy counselling enables screening of medication for teratogenicity. First trimester exposure to angiotensin-converting enzyme (ACE) inhibitors leads to an increase in congenital malformation, and exposure in the second and third trimesters leads to a recognised fetopathy [7,27]. Angiotensin receptor blockade (ARB) is also associated with adverse fetal effects [28]. Over one quarter of the women with CKD who attended for pre-pregnancy counselling were prescribed either ACE inhibitors and/or ARBs. Counselling with regard to the timing of drug discontinuation and instruction for requantification of proteinuria, represented a considerable component of the pre-pregnancy counselling process.

Approximately one fifth of women were taking mycophenolate mofetil, which is also a recognised teratogen [29]. Substitution with azathioprine and confirmation of stable disease is recommended prior to pregnancy [3]. Data concerning the maternal renal consequences of this drug replacement are reassuring in both renal transplantation and women with stable lupus nephritis [30].

Despite recommendations for pre-pregnancy counselling, there are limited data regarding the optimum way to deliver this service. The multidisciplinary model presented here includes a consultant obstetric physician, obstetrician and nephrologist offering counselling to women and their partners. The questionnaire identified that women were satisfied with this process and the provision of information. Subjective levels of understanding were excellent and decision-making regarding pregnancy was facilitated for the majority. Access was however highlighted by the women. Pathways into the clinic, and to other specialists, need to be examined in order to ensure that individualised pre-pregnancy care is achieved for all women according to demand.

17% of the women found the pre-pregnancy counselling process intimidating in the format of a ‘panel’ of three doctors. This structure contrasts with other multidisciplinary models in the UK. For example the well-established multidisciplinary format of cancer care involves relevant professions meeting in the absence of the patient aiming to facilitate unbiased discussion [31]. This model removes some elements that are likely to contribute to a feeling of intimidation. However, patient autonomy is dependent on the clarity, consistency and completeness of information, and the cancer model does have recognised deficiencies in patient communication and the provision of information [32]. Our multidisciplinary model allows women’s views and preferences to explicitly inform decision-making and promotes autonomy via the provision of an immediate resource of expertise.

At St. Thomas’ Hospital, antenatal care for women with CKD is provided by the same clinicians who deliver pre-pregnancy counselling. Anecdotally, it was felt that having prepared women for the possibility of adverse outcomes, women who had received pre pregnancy counselling were not as shocked or upset when complications arose as those who had not received counselling. In addition, content analysis of the questionnaire free text data revealed that three women (4% of responding cohort) had opted not to attempt a pregnancy following pre-pregnancy counselling. These factors reveal the importance of a risk assessment for women with CKD contemplating pregnancy. The communication of risk can reduce the incidence of complications if women opt not to conceive, as well as the psychological impact if complications do occur. However, the patient’s emotional response to the consultation needs to be acknowledged and supported.

Conclusion from this study is limited by a response rate of only 40%, which means that responder bias cannot be excluded. In addition, recall bias is a factor, especially for those women who attended clinic in the earlier years of the study. Qualitative research within the field of pre-pregnancy counselling is limited and validated questionnaires do not exist in published literature. The questionnaire used in this study was piloted prior to use, and underpinned by an established framework of patient-centred care. However, the structured design with categorical responses may not have allowed women to express fully their experience, and the questionnaire may therefore lack validity. Although free text was invited and did show consensus with the discrete data, this was not completed by all respondents. Further development and validation of a measure to assess the experience of pre-pregnancy care for women with CKD would be valuable. Mixed method research including qualitative methods, would help to define domains of the pre-pregnancy counselling experience from the patient’s perspective, thereby validating and informing future research within this field.

This study confirms that adverse pregnancy outcomes in women with CKD are more common than tertiary hospital controls. A limitation of the study was establishing a formal diagnosis of pre-eclampsia, which is challenging in both clinical and research settings [33], particularly in women with CKD. Furthermore, hospital databases frequently under-report this complication [34]. Despite this, the incidence of pre-eclampsia in our cohort was high (20%). Preterm delivery (<37 weeks) occurred in half of the women with CKD Stages 3A or 3B and approximately one in seven pregnancies was lost after the first trimester. Data were only available for deliveries at St. Thomas’ Hospital and referral bias is possible, with high risk women being advised to book for tertiary level antenatal care, and women with complications being transferred in antenatally. However, when compared to a control population with non-renal comorbidity also delivering at St. Thomas’ Hospital over the same time period; rates of pre-eclampsia, intrauterine growth restriction, preterm birth, intrauterine death and mid-trimester miscarriage were significantly higher for women with CKD. In addition, outcome data from this study are consistent with other series, which show comparable rates of pre-eclampsia (22%), preterm delivery (30%) and fetal growth restriction (25%) in women with a serum creatinine >125 μmol/l, which equates roughly to CKD Stage 3 [1].

Another limitation of the study is the lack of a formal comparison between those who have and have not attended a pre-pregnancy counselling clinic. However, a randomized study would not be ethical and a robust retrospective evaluation of whether women had received counselling prior to pregnancy by a different team is not possible. It is possible that pre–pregnancy counselling may prevent some adverse outcomes by ensuring optimisation of pre-pregnancy blood pressure and replacement of teratogenic medications, and this should be studied prospectively.

## Conclusions

There is a growing requirement for renal pre-pregnancy counselling services and this is the first study of acceptability and the impact from the perspective of women with CKD. This study offers evidence that a specialist clinic provides high levels of patient satisfaction. Due to advances in medical care, complex renal diagnoses exist with increasing frequency in women of childbearing age. Hypertension, proteinuria and teratogenic medications are prevalent in these women and need to be optimised before pregnancy. In addition, maternal and neonatal adverse outcomes are common in pregnant women with CKD and shared-decision making is only possible if that risk is understood and communicated effectively. This study provides proof of concept that there is a need for and value to pre-pregnancy counselling in allowing women to make an informed decision regarding pregnancy as well as preparing them for possible and likely complications.

## Additional files

Additional file 1:
**Patient experience questionnaire.**
Additional file 2:
**Annual number pre-pregnancy counselling appointments (2003–2011 = 187 attendances for 179 women).**
