# Supplementary material for: Pre-pregnancy counselling for women with chronic kidney disease: a retrospective analysis of nine years’ experience
Source: BMC Nephrol. 2015 Mar 14;16:28. doi: 10.1186/s12882-015-0024-6 (PMC4377018; doi:10.1186/s12882-015-0024-6)
Supplement: Additional file 1: — Patient experience questionnaire. [file 12882_2015_24_MOESM1_ESM.doc]

Additional file 1: Table S1 Patient experience questionnaire

| Questionnaire components of patient satisfaction |
| --- |
| - With regards to the possible risks of pregnancy, did you find the clinic informative? - Do you feel that you and your partner were given the opportunity to raise any concerns you had? - Did you feel that the doctors listened to your concerns and those of your partner? - Did you understand the advice given to you by the doctors? - Do you think that your visit to the clinic and the advice given helped you reach a decision about whether to proceed with a pregnancy? - Did you receive a copy of the letter written about your visit to the clinic? - Did you find this letter useful? - Did you find the presence of lots of doctors frightening or intimidating? - Was it helpful to have three consultants (nephrologist (kidney specialist), obstetrician and obstetric medicine physician) present at the same time? |
| Four-point answer scale |
| - Yes, definitely - Yes, probably - No, probably not - Definitely not |
