# Supplementary material for: Pre-pregnancy counselling for women with chronic kidney disease: a retrospective analysis of nine years’ experience
Source: BMC Nephrol. 2015 Mar 14;16:28. doi: 10.1186/s12882-015-0024-6 (PMC4377018; doi:10.1186/s12882-015-0024-6)

Additional file 2: Figure S1 Annual number pre-pregnancy counselling appointments (2003-2011 = 187 attendances for 179 women)
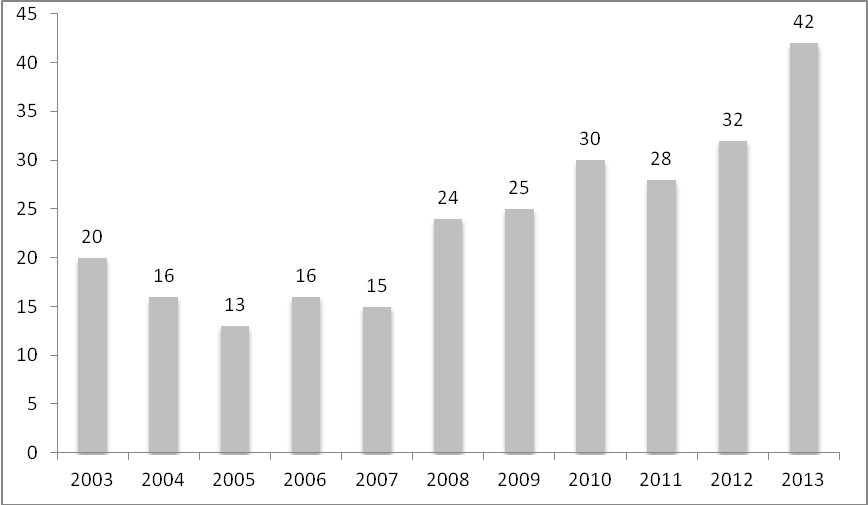

Supplement: Additional file 2: — Annual number pre-pregnancy counselling appointments (2003–2011 = 187 attendances for 179 women). [file 12882_2015_24_MOESM2_ESM.doc]
